# Computational analysis of a novel mutation in *ETFDH *gene highlights its long-range effects on the FAD-binding motif

**DOI:** 10.1186/1472-6807-11-43

**Published:** 2011-10-21

**Authors:** Tze-Kiong Er, Chih-Chieh Chen, Yen-Yi Liu, Hui-Chiu Chang, Yin-Hsiu Chien, Jan-Gowth Chang, Jenn-Kang Hwang, Yuh-Jyh Jong

**Affiliations:** 1 Division of Molecular Diagnostics, Department of Laboratory Medicine, Kaohsiung Medical University Hospital, Kaohsiung Medical University, 100 Shih-Chuan 1st Rd., Kaohsiung, 80708, Taiwan; 2 Graduate Institute of Medicine, College of Medicine, Kaohsiung Medical University, 100 Shih-Chuan 1st Rd., Kaohsiung, 80708, Taiwan; 3 Institute of Bioinformatics and Systems Biology, National Chiao Tung University, 75 Bo-Ai Street, Hsinchu, 30068, Taiwan; 4 Department of Medical Genetics and Pediatrics, National Taiwan University Hospital, 8 Chung-Shan South Road, Taipei, 10041, Taiwan; 5 Department of Pediatrics, Kaohsiung Medical University Hospital, Kaohsiung Medical University, 100 Shih-Chuan 1st Rd., Kaohsiung, 80708, Taiwan; 6 Institute of Clinical Medicine, College of Medicine, Kaohsiung Medical University, 100 Shih-Chuan 1st Rd., Kaohsiung, 80708, Taiwan; 7 Center for Excellence in Environmental Medicine, Kaohsiung Medical University, 100 Shih-Chuan 1st Rd., Kaohsiung, 80708, Taiwan

## Abstract

**Background:**

Multiple acyl-coenzyme A dehydrogenase deficiency (MADD) is an autosomal recessive disease caused by the defects in the mitochondrial electron transfer system and the metabolism of fatty acids. Recently, mutations in electron transfer flavoprotein dehydrogenase (*ETFDH*) gene, encoding electron transfer flavoprotein:ubiquinone oxidoreductase (ETF:QO) have been reported to be the major causes of riboflavin-responsive MADD. To date, no studies have been performed to explore the functional impact of these mutations or their mechanism of disrupting enzyme activity.

**Results:**

High resolution melting (HRM) analysis and sequencing of the entire *ETFDH *gene revealed a novel mutation (p.Phe128Ser) and the hotspot mutation (p.Ala84Thr) from a patient with MADD. According to the predicted 3D structure of ETF:QO, the two mutations are located within the flavin adenine dinucleotide (FAD) binding domain; however, the two residues do not have direct interactions with the FAD ligand. Using molecular dynamics (MD) simulations and normal mode analysis (NMA), we found that the p.Ala84Thr and p.Phe128Ser mutations are most likely to alter the protein structure near the FAD binding site as well as disrupt the stability of the FAD binding required for the activation of ETF:QO. Intriguingly, NMA revealed that several reported disease-causing mutations in the ETF:QO protein show highly correlated motions with the FAD-binding site.

**Conclusions:**

Based on the present findings, we conclude that the changes made to the amino acids in ETF:QO are likely to influence the FAD-binding stability.

## Background

Multiple acyl-CoA dehydrogenase deficiency (MADD), also known as glutaric aciduria type II, is an autosomal recessively inherited disorder of fatty acid metabolism [1]. In the majority of cases, the disorder is caused by a defect in either the alpha- or beta-subunit of the electron transfer flavoprotein (*ETFA*; OMIM 608053, *ETFB*; OMIM 130410) or the electron transfer flavoprotein dehydrogenase (*ETFDH*; OMIM 231675) genes. However, in some patients, the disorder may result from a few, currently unidentified, disruptions to riboflavin metabolism [2-4]. Because of a deficiency of *ETF *or *ETFDH*, the acyl-CoA dehydrogenases are unable to transfer the electrons generated by the dehydrogenation reactions. This results in the accumulation of various intramitochondrial acyl-CoA esters. Recently, *ETFDH *mutations were reported to be major causes of riboflavin-responsive MADD [5].

Characteristics such as organic aciduria may be detected only during periods of illness or catabolic stress. In the majority of cases, depending on the metabolic status of the patient, plasma acylcarnitine analysis may not provide a definitive diagnosis. Amongst the most daunting challenges facing MADD is metabolic heterogeneity, in particular, for the diagnosis of MADD. Thus, numerous molecular diagnostic methods have been established for analyzing the *ETF *and/or *ETFDH *genes for the purpose of achieving the final diagnosis of MADD. Previously, we reconfirmed the high prevalence of the c.250G > A (p.Ala84Thr) mutation in Taiwanese patients with riboflavin-responsive MADD, as well as in the normal population using a high-resolution melting (HRM) analysis [6]. It should be noted that all the nine patients examined, including three pairs of siblings, harbor this mutation and four were homozygous. We estimated the allele frequency to be 0.004 for the p.Ala84Thr mutant (c.250G > A). Thus, the estimated carrier frequency of this mutation in the Taiwanese population is 1:125 (0.8%). The homozygous mutant frequency was 1:62,500. Nevertheless, the carrier frequency of c.250G > A is estimated to be 1.35% amongst the population in southern China. Thus, ~1:22,000 Han Chinese are expected to suffer from riboflavin-responsive MADD [7]. Intriguingly, p.Ala84Thr has been identified in Asian countries including Taiwan, Hong Kong, Singapore, Thailand and southern China [6-11]. A total of 69 patients were examined from the above referenced literature and diagnosed with late-onset MADD. Amongst these patients, the mutation c.250G > A in the *ETFDH *gene was detected in 49 patients with homozygous mutations and 20 patients with heterozygous mutations. To date, this mutation has never been reported in Western countries [5]. Thus, it has been suggested that the most common mutation p.Ala84Thr is ethnic-specific. In addition, the mutation of p.Ala84Thr most likely accounts for the mild phenotype and response to riboflavin. Here, a patient presented at 11/2 years represented a mild to late-onset MADD and the patient slightly improved following riboflavin treatment (Additional file 1, Table S1). The patient had compound heterozygous mutations (p.Ala84Thr/p.Phe128Ser) of the *ETFDH *gene. Currently, there is no related study to explore the underlying mechanism for the most common mutation (p.Ala84Thr).

Flavin adenine dinucleotide (FAD) is a cofactor for the electron transfer flavoprotein:ubiquinone oxidoreductase (ETF:QO; encoded by the *ETFDH *gene) which constitutes the electron-transport pathway for a number of mitochondrial flavoprotein dehydrogenases that are mainly involved in fatty-acid and amino-acid metabolism. Riboflavin (the precursor of FAD) treatment has been shown to strikingly ameliorate the symptoms and metabolic profiles in MADD patients [1,4,6,12]. This may enhance the conformational stabilization of the mutant ETF:QO protein [13]. Two of the mutations (p.Ala84Thr and p.Phr128Ser) are located in the FAD-binding domain; however, the two amino acids do not have direct interactions with FAD according to the predicted 3D structure of ETF:QO. The closest distances between the mutations (p.Ala84Thr and p.Phr128Ser) and the FAD binding residues are 8.6 and 13.6 Å, respectively. Therefore, to explore the effects of the mutations on ETF:QO dynamics, molecular dynamics (MD) simulations of the wild type (WT) and mutant type (MT) ETF:QO in the same model environment were compared. Besides the MD simulations, an alternative method, normal mode analysis (NMA), for studying protein motions was used to analyze the dynamic correlations between the mutation sites and the FAD-binding motif.

In this study, molecular modeling, MD simulations and NMA were performed to investigate the structural implications of the underlying mutations on the protein conformation. The obtained results may provide an insight into the pathomechanism of MADD.

## Methods

### Patients

The patient was a girl aged 11/2 years who was clinically diagnosed with MADD in 2009. Her dried-blood spot sample was sent for acylcarnitine analysis. The results demonstrated elevation of acylcarnitines during the illness, consistent with glutaric aciduria type 2. Gas chromatography-mass spectrometry revealed significant elevations of urinary glutaric acid, 3-methylglutarate, 3-methylcluconate, adipate, 3-methyladipate, octenedioate, suberate, and hexanoylglycine. The present study was approved by the Institutional Review Board (IRB) of Kaohsiung Medical University Hospital.

### Genetic analysis

The blood samples were collected from the patient and the family members as well as from normal controls with informed consents. The genomic DNA samples were extracted from peripheral whole blood using the NucleoSpin^® ^Blood Kit (Macherey-Nagel), according to the manufacturer's instructions.

### Identification of mutations

HRM analysis was applied for screening the mutation of *ETFDH*, as previously described [6]. PCR was carried out in duplicate in a 10 μL final volume using the LightCycler ^® ^480 High-resolution Melting Master (Reference 04909631001, Roche Diagnostics) 1 × buffer which contained Taq polymerase, nucleotides, the dye ResoLight and 30 ng DNA. The primers and MgCl_2 _were used at a concentration of 0.25 μM and 2.5 mM, respectively for identifying the *ETFDH *gene mutations.

The HRM assays were conducted using the LightCycler^® ^480 instrument (Roche Diagnostics) provided with the software LightCycler^® ^480 Gene-Scanning Software Version 1.5 (Roche Diagnostics).

The PCR program required a SYBR Green I filter (533 nm). The program consisted of an initial denaturation-activation step at 95°C for 10 min, followed by a 45-cycle program. The 45-cycle program consisted of the following steps: denaturation at 95°C for 15 s, annealing at 58°C or 60°C for 15 s and elongation at 72°C for 15 s with the reading of the fluorescence. The acquisition mode was single. The melting program consisted of three steps: denaturation at 95°C for 1 min, renaturation at 40°C for 1 min, and the subsequent melting that included continuous fluorescent readings from 60 to 90°C at a rate of 25 acquisitions per degree centigrade.

To confirm the results of the HRM analysis, a sequencing analysis was also performed for all the samples. After the HRM analysis, the samples were purified using the FavorPrep™ PCR clean-up mini kit. The PCR products generated after HRM were sequenced directly. The sequence reaction was performed in a final volume of 10 μL, comprising 5 μL of the purified PCR product, 2.5 μM of each PCR primer and 1 μL of the ABI PRISM terminator cycle sequencing kit v1.1 (Applied Biosystems). The sequencing program started from 96°C for 1 min and then followed by 25-cycle PCR program. The program consisted of the following steps: denaturation at 96°C for 10 s; annealing at 50°C for 5 s; and elongation at 60°C for 4 min. The sequence detection was performed using the ABI Prism 3130 Genetic Analyzer (Applied Biosystems). To ensure that the mutation found in the patient was the one specifically present in patients with MADD, 60 healthy control chromosomes were also screened.

### Sorting intolerant from tolerant (SIFT) analysis

SIFT tool [14-16] generates multiple alignments of the sequences over different species to look at the conserved sequence of a gene; it asses the conserved amino acid positions and analyzes the effect of missense changes on the conserved structure of proteins over the course of evolution. The SIFT tool assigns a score to the mutations, and the score of < 0.05 is considered potentially damaging.

### Structure prediction of human wild type (WT) and mutant type (MT) ETF:QO

Three-dimensional (3D) structures of the human ETF:QO were predicted using the (PS)^2 ^server [17,18]. (PS)^2 ^automatically uses an effective consensus strategy. The above mentioned strategy combines structural- [18] and profile-based [19-21] comparison methods for both template selection and target-template alignment. (PS)^2 ^server selected the porcine ETF:QO (PDB entry: 2GMH) [22] as the model template. The ETF:QO models were built based on a sequence and structural alignment from the above mentioned template. A total of six models were yielded for each target by using a set of parameters. These models were based on the alternative target-template alignments. The qualities of the modeled structures were checked using Ramachandran plots [23]. The model with the highest quality was selected as the final predicted structure for the target. Finally, the protein-ligand complex was constructed by superimposing the predicted human ETF:QO model to the 3D structure of the porcine ETF:QO-ligand complex.

### Molecular dynamics simulations

Molecular dynamics (MD) simulations were performed using the software GROMACS ver. 4.5.1 [24]. OPLS-AA all-atom force field was used for the energy calculations. The models were solvated with simple point charge (SPC) water molecules, and the models were simulated in a triclinic box [25] with periodic boundary conditions. The simulations were performed in the canonical NVT (number of particles, volume and temperature) ensemble. The energy of the models was first minimized using the steepest descent algorithm. This was followed by the position-restrained MD simulations for 200 ps. The linear constraint solver (LINCS) algorithm was used to constrain the bonds [26]. The MD simulations were carried out at constant pressure and temperature for 2.0 ns using an integration time step of 2 fs. The non-bonded interactions were cutoff at 10 Å. At every 100 time steps, the coordinates from the MD simulations were saved.

### Analysis of MD trajectories

The objective was to understand the structural and functional implications of the amino acid substitution. Thus, the trajectories of WT and MT were analyzed for the following structural properties as a function of time: a) the root mean square deviation (RMSD) of the Cα atoms with respect to the starting conformation; b) RMSD of the FAD-binding motif with respect to the starting conformation; and c) the B-factors [27] of the Cα atoms which were calculated from the last 1 ns of the MD trajectories. The B-factors in protein structures reflect the fluctuation of atoms about their average positions. A large B-factor indicates high flexibility of individual atoms.

### Normal mode analysis

Normal mode analysis (NMA) is a powerful tool to estimate the dynamics based on structure [28-31]. Low-frequency normal modes describing the large-scale real-world motions of a protein have been demonstrated to be related to biological function [28,32]. In the present study, the NMA was performed using the software GROMACS ver. 4.5.1 by diagonalization of the mass-weighted Hessian matrix. The force field-based NMA requires careful energy minimization of the initial structure. We first minimized the human ETF:QO structure with the steepest descent integrator for a couple of steps. Next, the L-BFGS integrator was used to minimize the maximum force close to zero and subsequently generate a Hessian matrix. The NMA was performed using the algorithm in GROMACS. Finally, the pair-wise residue correlation was calculated. The above mentioned correlation was calculated as the average correlation summed over 50 lowest nonzero frequency normal modes [33].

## Results

### Patient and molecular diagnostic

**Figure 1:**
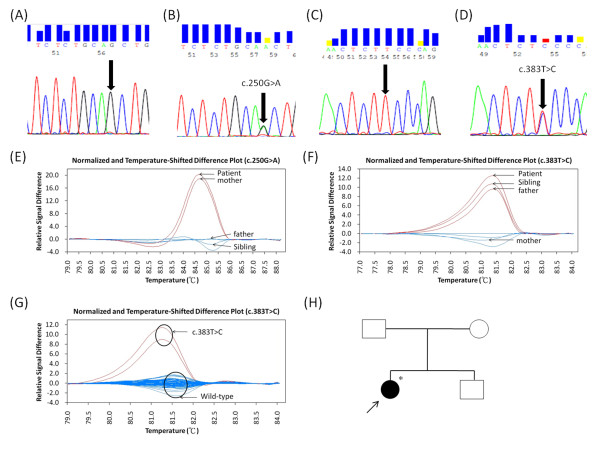
**HRM analysis of *ETFDH *gene**. Electropherograms of various mutations: (A) c.250G > A (wild type); (B) c.250G > A (heterozygous); (C) c.383T > C (wild type); (D) c.383T > C (heterozygous); (E) Normalized and temperature-shifted difference plots, the melting profile of c.250G > A; (F) Normalized and temperature-shifted difference plots, the melting profile of c.383T > C; (G) The mutation of c.383T > C was not detected in 60 unrelated control chromosomes (wild type); (H) Affected pedigree with familial segregation of both mutations. Squares, male subjects; circles, female subjects. Affected and unaffected subjects are represented by solid and open symbols, respectively. The subject with a sample available for the present study is indicated with an asterisk.

HRM analysis of *ETFDH *gene revealed a novel T > C point mutation at position 383. The point mutations were confirmed by direct DNA sequencing (Figures 1A-1D). The hotspot mutation c.250G > A (p.Ala84Thr) (Figures 1A and 1B) and a novel mutation c.383T > C (p.Phe128Ser) (Figures 1C and 1D) in the *ETFDH *gene were identified in this patient. These mutations are highly conserved amongst the different species (Additional file 2, Table S2). The SIFT tool analysis revealed a score of < 0.05 and predicted that the replaced amino-acid was potentially damaging. This indicates that these mutations are most likely pathogenic. Figures 1E and 1F show the melting profiles of the *ETFDH *mutations in the patient's family. The patient had compound heterozygous mutations (p.Ala84Thr/p.Phe128Ser). The sibling was a carrier of c.383T > C (p.Phe128Ser). The father and mother were carriers of c.383T > C (p.Phe128Ser) in exon 3 and c.250G > A (p.Ala84Thr) in exon 3, respectively (Figures 1E and 1F). The above mentioned mutation was not detected in the 60 unrelated control chromosomes (wild type) (Figure 1G). The pedigree is presented in Figure 1H.

### Structure prediction of human ETF:QO

**Figure 2:**
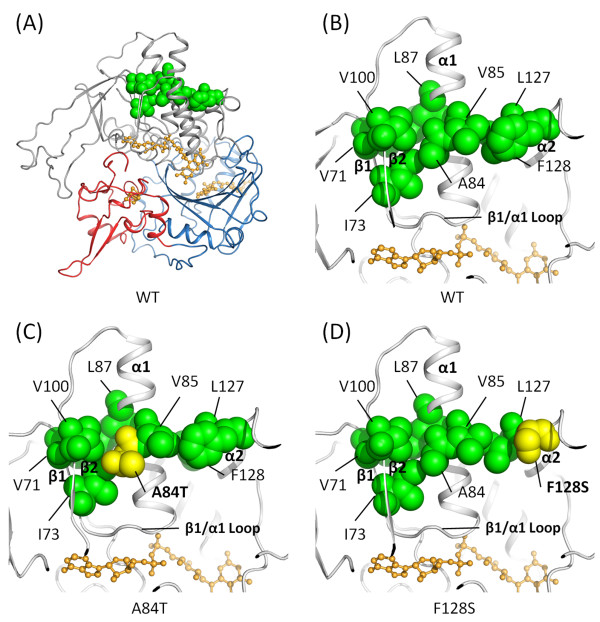
**Structure prediction of ETF:QO**. (A) The predicted wild-type model of human ETF:QO. The structure comprises three domains: the FAD-binding domain (gray), the ubiquinone-binding domain (blue) and the 4Fe4S cluster domain (red). The three redox centers: FAD, ubiquinone and the 4Fe4S cluster are shown in ball-and-stick representations. A cluster of hydrophobic residues which are located at the FAD-binding domain and at a distance below 4 Å around the mutation residues (p.Ala84Thr and p.Phe128Ser) are depicted in the sphere model. (B) A close-up view of the hydrophobic residues (V71, I73, A84, V85, L87, V100, L127 and F128). The α-helices and β-strands are also labeled. (C) Model of the p.Ala84Thr mutant in which the hydrophobic residue A84 is replaced by the hydrophilic residue threonine (yellow). (D) Model of the Phe128Ser mutant in which the hydrophobic residue F128 is replaced by the hydrophilic residue serine (yellow). The 3D molecular graphs are displayed using PyMOL [51].

Human ETF:QO models were constructed using the published porcine ETF:QO crystal structure (PDB Id: 2GMH) as the template (Figure 2A). A close-up view of the hydrophobic residues which are located at the FAD-binding domain, including V71, I73, A84, V85, L87, V100, L127, and F128 is shown in Figure 2B. The FAD-binding residues are located at the loop that connects strand β1 and helix α1. Therefore, it is reasonable to hypothesize that these hydrophobic residues would most likely participate in hydrophobic interactions to stabilize FAD binding. The ETF:QO mutations of p.Ala84Thr and p.Phe128Ser were constructed in the same way as the WT structure. Both replacements are located at the center and peripheral of the hydrophobic patch, respectively (Figures 2C and 2D).

### Molecular dynamics simulations

For the MD simulations, the trajectories of the WT and MT ETF:QO in the explicit solvent were calculated. The backbone RMSD values for WT and MT ETF:QO during the production phase relative to the starting structures were plotted (Additional file 3, Figure S1). These values were plotted to obtain an estimate of the MD trajectory quality and convergence. For the simulations of WT and MT ETF:QO, the obtained data demonstrate that after a rapid increase during the first 0.2 ns, the trajectories are stable with average values of 1.43, 1.38 and 1.51 Å for WT and the p.Ala84Thr and p.Phe128Ser ETF:QO mutants, respectively. Statistical analysis of the RMSD data reveals that the trajectories are more stable after the first 1.0 ns. Therefore, only the second half of the trajectory was analyzed.

**Figure 3:**
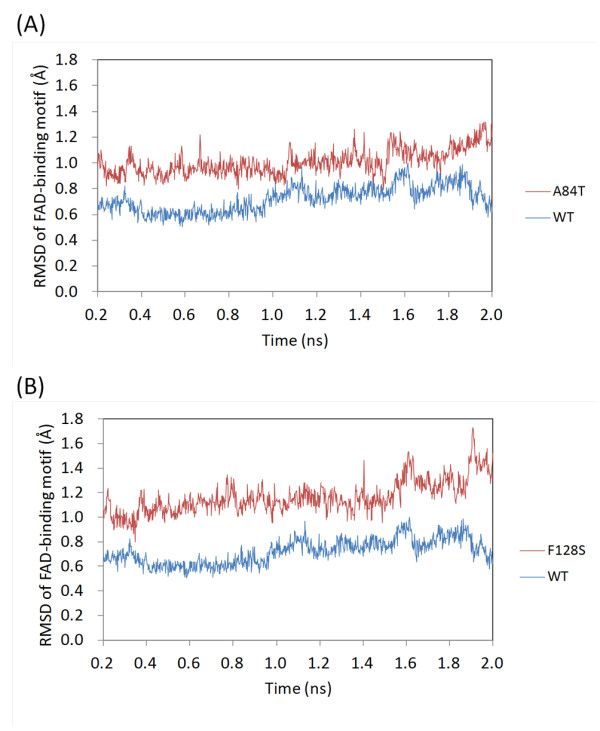
**RMSD plots of the FAD-binding motif**. Comparison of the RMSD plots of the FAD-binding motif (residues 71-90) of WT and MT (A) p.Ala84Thr and (B) p.Phe128Ser structures with respect to the starting conformation during the course of the simulation. WT and MT plots are presented in blue and red, respectively.

Based on Zhang's study [22], a segment corresponding to residues 71-90 of human ETF:QO was found to form the FAD-binding motif. The binding site was located at the β1/α1 loop (Figure 2B). The RMSD of the FAD-binding motif with respect to the starting conformation was compared for the WT and MT structures during the course of the simulations. The RMSD was found to increase as a function of time for the MT p.Ala84Thr (Figure 3A) and MT p.Phe128Ser (Figure 3B) when compared with the WT.

**Figure 4:**
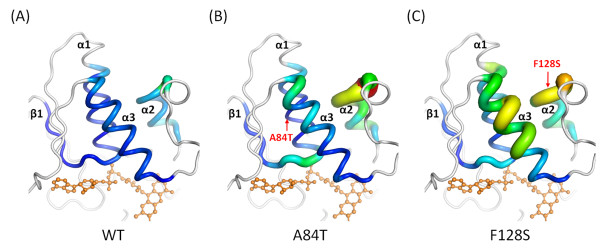
**B-factors analysis**. Structures of the (A) WT, (B) p.Ala84Thr and (C) p.Phe128Ser ETF:QO proteins are drawn in cartoon putty representation at the β1, α1, α2, and α3 regions, where the color is ramped by residue from blue as the lowest B-factor value to red as the highest B-factor value. In addition, the size of the tube also reflects the value of the B-factor, where the larger the B-factor the thicker the tube. The structures in the other regions are colored in white and displayed in cartoon tube representation, where the size of the tube is independent of the B-factors. The red arrows indicate the positions of the mutations.

The analysis of the B-factors for each residue at the β1, α1, α2, and α3 regions revealed that the atomic fluctuations of the p.Ala84Thr and p.Phe128Ser mutants were significant at the β1/α1 loop (involved in the FAD binding) when compared with the WT values (Figure 4). Helix α2, which may be involved in the support and stabilization of the α1 and α3 helices, was also found to have higher B-factors in both mutants (Figures 4B and 4C). Moreover, the fluctuations of the MT p.Phe128Ser were also observed to be significant at helix α3 (Figure 4C). From the above mentioned analysis, it was observed that the mutations of p.Ala84Thr and p.Phe128Ser may induce additional fluctuations in the β1/α1 loop, α2, and α3 regions. Such fluctuations may promote instability in this region of the protein and hamper FAD binding.

### Normal mode analysis

**Figure 5:**
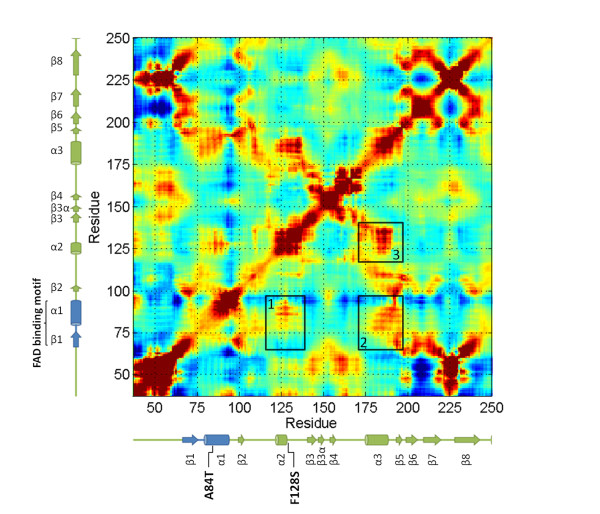
**Cross-correlation map calculated from the NMA**. A negative value refers to an anti-correlation between residue fluctuations (blue), whereas a positive value indicates concerted motion in the same direction (red).

The correlation map for ETF:QO is shown in Figure 5. General structural elements of ETF:QO can be identified by the pattern of the cross-correlations. The enlargements along the diagonal are typical for α-helices, where motions of amino acids adjacent in the sequence are correlated, such as the elements of the helices α1, α2 and α3 (Figure 5). Cross-correlations that extend as plumes from the diagonal correspond to anti-parallel elements of the secondary structure such as the strands β5, β6, β7 and β8 (Figure 5). The cross-correlations are also found between the multiple structural elements, whereby some result from specific contacts, while others are longer range in nature. For example, long-range cross-correlations are observed between the FAD-binding motif (colored in blue) and helix α2 (black square 1). Between the FAD-binding motif and helix α2, correlations exist because of the presence of a hydrophobic patch consisting of V85 (helix α1), L127 (helix α2) and F128 (helix α2) (Figure 2B). Helix α3 also displays significant correlated motion with the FAD-binding motif and helix α2 (black squares 2 and 3). These results suggest correlated dynamics between the FAD-binding motif, helix α2 and helix α3.

## Discussion

In this study, we found that the p.Ala84Thr and p.Phe128Ser mutations are most likely to alter the protein structure near the FAD binding site as well as disrupt the stability of the FAD binding required for the activation of ETF:QO using MD simulations and NMA. Interestingly, NMA revealed that several reported disease-causing mutations in the ETF:QO protein show highly correlated motions with the FAD-binding site.

In the FAD-binding family, the pyrophosphate moiety binds to the most strongly conserved sequence motif [22]. A subset of the proteins that adopt the Rossmann fold [34-36] also bind to the nucleotide cofactors such as FAD and NAD(P), and function as oxidoreductases. The Rossmann fold can often be identified by the short amino-acid sequence motif, GxGxxG. The pivotal role of the Gly residues in the conserved central GxGxxG is well understood [37,38]. The Rossmann fold begins with a β-strand connected by a short loop to an α-helix [39]. As previously described, the most conserved and well-studied sequence motif, xhxhGxGxxGxxxhxxh(x)_8_hxhE(D), is part of the Rossmann fold [37,40-42]. Here × is any residue and h is a hydrophobic residue. The hydrophobic residues provide hydrophobic interactions between the α-helix and the β-sheet. Intriguingly, the mutation of p.Ala84Thr is located at a conserved hydrophobic residue of this motif of the Rossmann fold (Figure 2C). In addition, the mutation of p.Phe128Ser is also located at this hydrophobic patch (Figure 2D). The above mentioned observations, along with the obtained results of the trajectory analyses (Figures 3 and 4), strongly suggest that these mutations could influence the structural stability of the FAD-binding motif. Thus, it could lead to unstable FAD binding. Therefore, we postulate that p.Ala84Thr and p.Phe128Ser may alter the stability of the FAD-binding site and hamper efficient binding of the ligand. In turn, this instability of the FAD-binding site may impair the activity of the electron-transport system.

The binding of FAD is important for the catalytic activity of flavoproteins as well as for the correct folding, assembly and protein stability [43-45]. A reduced availability of intra-mitochondrial FAD may result in a less stable or inactive conformation of the proteins. As a result, an accelerated breakdown could result. However, supplementation of riboflavin is most likely to increase the intra-mitochondrial FAD concentration. As a result, FAD binding could be promoted. This could ameliorate the effect of the mutations that reduce the affinity of ETF:QO for FAD [5,13]. It should be noted that riboflavin treatment has been shown to ameliorate the symptoms and metabolic profiles in recent studies [5,6,8,11,12].

If the charge or size of the amino acid is altered and the amino acid is buried within the core of the protein, folding may not proceed and the result is a complete loss of function. Such a loss of function has been found for a large number of variant short-chain acyl-CoA dehydrogenase (SCAD) and medium-chain acyl-CoA dehydrogenase (MCAD) proteins [46,47]. In addition, it may also cause conformational changes to the 3D structure by altering its hydrophobicity, charge, overpacking, or cavity at the buried site, leading to a temperate damage [48]. In the present study, we identified a novel mutation, p.Phe128Ser. We have used a NMA to characterize the detailed changes to protein fluctuations. The results suggest that the dynamics of the FAD-binding motif, helix α2 and helix α3 are correlated. The substitution of p.Phe128Ser may alter the hydrophobic interactions between helix α1 and helix α2 (Figure 5). It should be noted that numerous mutations near residue p.Phe128 have been identified. Wen *et al*. [48] recently identified three *ETFDH *mutations, p.Leu127Arg, p.Asp130Val, and p.Trp131Cys in riboflavin-responsive MADD patients in the north of China. Er *et al*. [6] and Liang *et al*. [8] reported one *ETFDH *mutation, p.Leu127His, in riboflavin-responsive MADD patients in the Taiwanese population. Law *et al*. [11] also reported one *ETFDH *mutation, p.Pro137Ser, in a Chinese family with riboflavin-responsive MADD. In addition, Goodman *et al*. [49] reported p.L138R as a disease-causing mutation. Interestingly, these mutations are located very close to each other in the region of the helix α2. Thus, these findings demonstrate the highly correlated motions of the FAD-binding motif (Figure 5 and Additional file 4, Figure S2). Previously recognized *ETFDH *mutations, p.Arg175Leu and p.Arg175His [6-8,50], were found in the region of helix α3. Both mutations have significantly correlated motions with the FAD-binding motif, as shown in Figure 5. Based on the present findings, we believe that the mutations to the amino acids in these regions are likely to affect the FAD-binding stability. Therefore, it is highly recommended that the *ETFDH *mutation in exon 3, 4 and 5 be initially screened. However, based on the B-factors analysis, the atomic fluctuations of the p.Leu127Arg, and p.Arg175His were not significant at the β1/α1 loop, which was involved in FAD binding (Additional file 5, Figure S3). According to published literatures, these MADD patients are compound heterozygous for two mutations. Therefore, it appears probable that other mutations most likely account for the disease state. Characterization of additional mutations in the other region of ETF:QO will be established in future studies. Currently, the present study is the first to demonstrate through NMA that several previously reported *ETFDH *gene mutations have highly correlated motions with the FAD-binding site.

## Conclusions

The present study is the first demonstration of p.Ala84Thr and p.Phe128Ser by molecular modeling, MD simulations, and correlated motion analysis. Along with the obtained results of the MD simulations and NMA, the present findings strongly suggest that p.Ala84Thr and p.Phe128Ser are likely to alter the ETF:QO protein structure near the FAD-binding site as well as disrupt the stability of FAD binding, which is essential for the activation of ETF:QO. The change of hydrophobic interaction may destabilize FAD binding, giving rise to a less stable or inactive conformation. Overall, the obtained findings have implications for the pathogenic mechanism of riboflavin-responsive MADD.

## Competing interests

The authors declare that they have no competing interests.

## Authors' contributions

Concept and design of the experiments: TKE, CCC, YYL, HCC, JGC, JKH and YJJ. Performance of the experiments: TKE and CCC. Data analysis and discussion: TKE, CCC, YYL, HCC, YHC, JGC, JKH and YJJ. Contribution of reagents/materials/analysis tools: HCC, YHC, JGC and JKH. Manuscript preparation: TKE, CCC, JGC, JKH and YJJ. All authors read and approved the final manuscript.

## Supplementary Material

Additional file 1 **Table S1**. Dried blood spot acylcarnitine profile measured using tandem mass spectrometry in the patient before and after riboflavin treatment. Click here for file

Additional file 2 **Table S2**. The highlighted amino acid alanine (A) and phenylalanine (F) shown in yellow and pink, respectively, at positions 84 and 128 are conserved in all the orthologs. Click here for file

Additional file 3 **Figure S1**. RMSD plots of the whole WT and MT (A) p.A84T and (B) p.F128S structures with respect to the starting conformation during the course of the MD simulations. Click here for file

Additional file 4 **Figure S2**. Structure of ETF:QO with the positions of the identified amino acid changes near residue F128 in riboflavin-responsive MADD patients labeled. Click here for file

Additional file 5 **Figure S3**. Structures of the WT and MT ETF:QO are drawn in cartoon putty representation. The red arrows indicate the positions of the mutations. Click here for file
