# Supplementary material for: Computational analysis of a novel mutation in ETFDH gene highlights its long-range effects on the FAD-binding motif
Source: BMC Struct Biol. 2011 Oct 21;11:43. doi: 10.1186/1472-6807-11-43 (PMC3209457; doi:10.1186/1472-6807-11-43)
Supplement: Additional file 1 — Table S1. Dried blood spot acylcarnitine profile measured using tandem mass spectrometry in the patient before and after riboflavin treatment. [file 1472-6807-11-43-S1.PDF]

Table S1. Dried blood spot acylcarnitine profile measured using tandem mass spectrometry in the patient before and after riboflavin treatment.

**Acylcarnitine**	**Before** (μM) 2009.09.07	**After** (μM) 2011.05.17	**Normal Range (0~5 years old)**
Acetylcarnitine (C2)	35.44	21.34	14.3
Butyrylcarnitine (C4)	0.9	0.49	0.4
Hexanoylcarnitine (C6)	1.03	0.4	0.185
Octanoylcarnitine (C8:1)	0.16	0.19	0.244
Decanoylcarnitine (C10:1)	0.34	0.26	0.151
Dodecanoylcarnitine (C12)	0.75	0.42	0.297
Tetradecenoylcarnitine (C14)	0.82	0.51	0.31
Tetradecenoylcarnitine (C14:1)	0.96	0.34	0.192
Palmitylcarnitine (C16)	1.82	1.06	1.936
Stearylcarnitine (C18)	1.38	0.68	0.825
Oleylcarnitine (C18:1)	1.94	0.93	1.221
