# Supplementary material for: Computational analysis of a novel mutation in ETFDH gene highlights its long-range effects on the FAD-binding motif
Source: BMC Struct Biol. 2011 Oct 21;11:43. doi: 10.1186/1472-6807-11-43 (PMC3209457; doi:10.1186/1472-6807-11-43)
Supplement: Additional file 2 — Table S2. The highlighted amino acid alanine (A) and phenylalanine (F) shown in yellow and pink, respectively, at positions 84 and 128 are conserved in all the orthologs. [file 1472-6807-11-43-S2.PDF]

Table S2. The highlighted amino acid alanine (A) and phenylalanine (F) shown in yellow and pink, respectively, at positions 84 and 128 are conserved in all the orthologs.

	p.Ala84Thr	p.Phe128Ser
Homo sapiens	GLSAAVRLKQLAVAH	KELFPDWKEKGAPLNTP
Bos taurus	GLSAAARLKQLAAQH	QELFPDWKEKGAPLNTP
Rattus norvegicus	GLSAAIRLKQLAAEQ	KELFPDWKEKGAPLNTP
Pongo abelli	GLSAAVRLKQLAAAH	KELFPDWKEKGAPLNTP
Xenopus laevis	GLSAAVRLKQLAAEC	EELFPDWKERGAPLNTP
Rattus norvegicus	GLSAAIRLKQLAAEQ	KELFPDWKERGAPLNTP
Mus musculus	GLSAAIRLKQLAAEQ	KELFPDWKEKGAPLNTP
