# Supplementary figures and images for: Computational analysis of a novel mutation in ETFDH gene highlights its long-range effects on the FAD-binding motif
Source: BMC Struct Biol. 2011 Oct 21;11:43. doi: 10.1186/1472-6807-11-43 (PMC3209457; doi:10.1186/1472-6807-11-43)

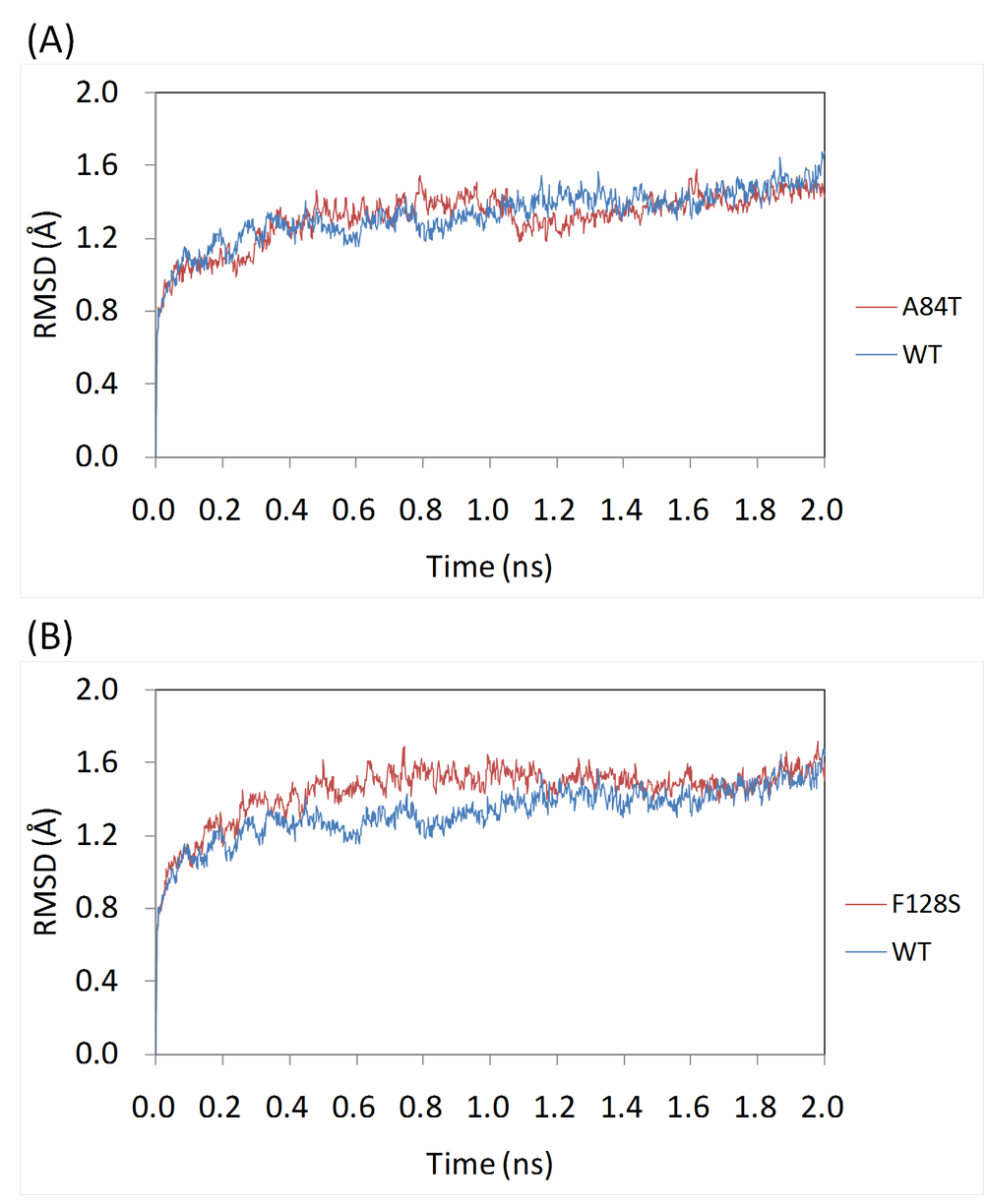

Supplement: Additional file 3 — Figure S1. RMSD plots of the whole WT and MT (A) p.A84T and (B) p.F128S structures with respect to the starting conformation during the course of the MD simulations. [file 1472-6807-11-43-S3.TIFF]

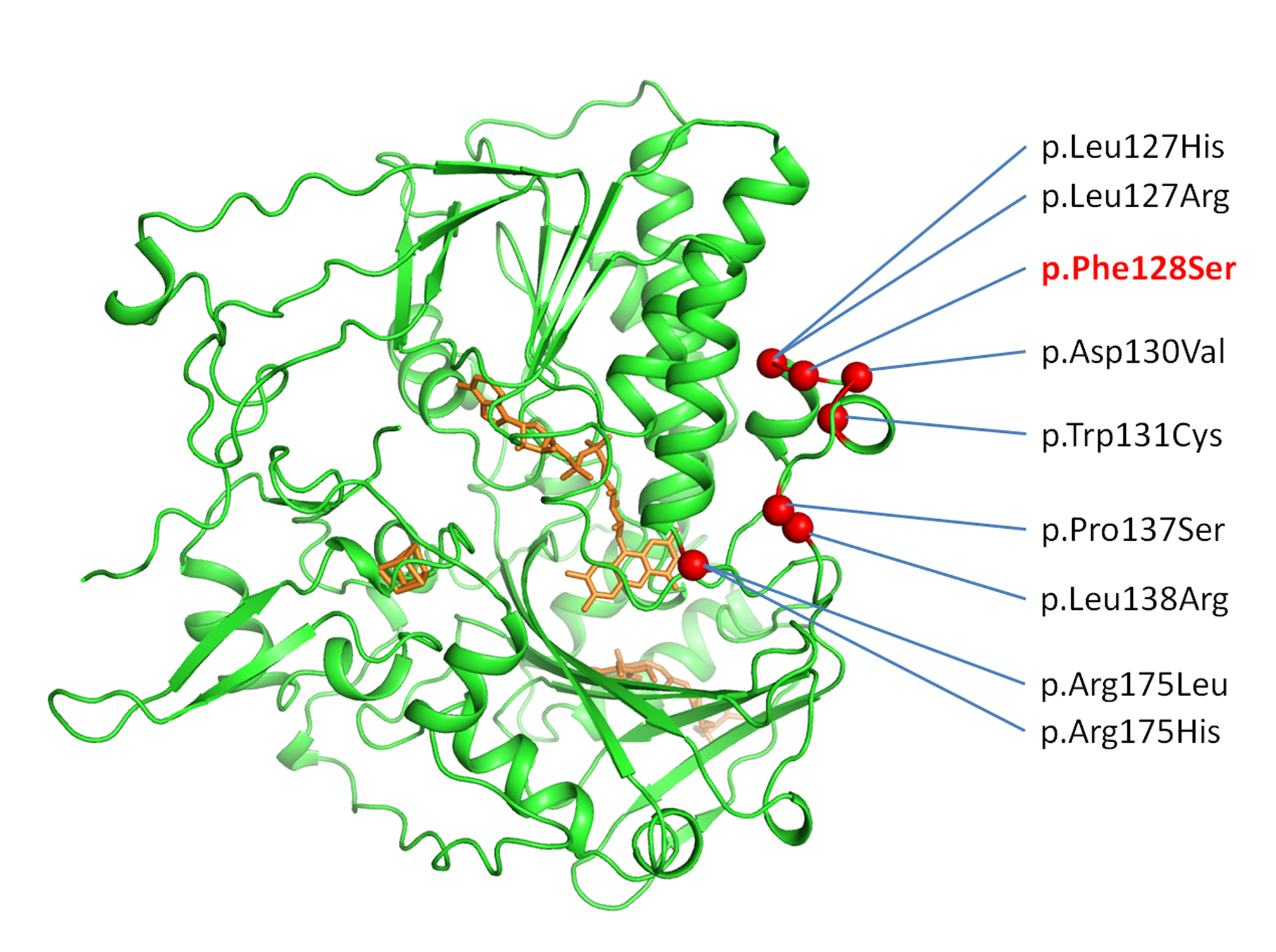

Supplement: Additional file 4 — Figure S2. Structure of ETF:QO with the positions of the identified amino acid changes near residue F128 in riboflavin-responsive MADD patients labeled. [file 1472-6807-11-43-S4.TIFF]

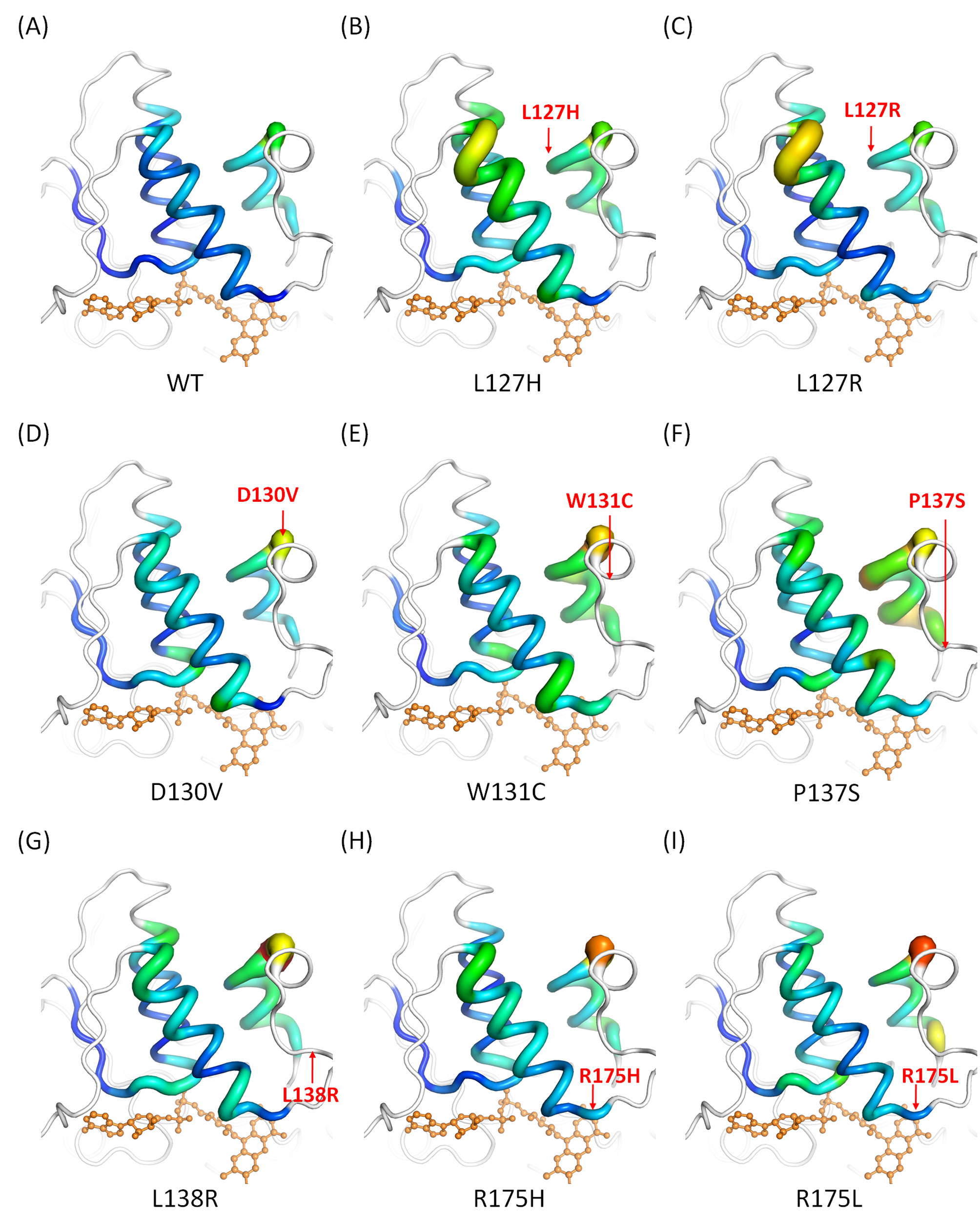

Supplement: Additional file 5 — Figure S3. Structures of the WT and MT ETF:QO are drawn in cartoon putty representation. The red arrows indicate the positions of the mutations. [file 1472-6807-11-43-S5.TIFF]
